# *Acinetobacter baumannii*: Role in Blood Stream Infection in Neonatal Unit, Dr. Cipto Mangunkusumo Hospital, Jakarta, Indonesia

**DOI:** 10.1155/2013/180763

**Published:** 2013-10-28

**Authors:** Enty Tjoa, Lucky Hartati Moehario, Andriansjah Rukmana, Rinawati Rohsiswatmo

**Affiliations:** ^1^Department of Microbiology, Faculty of Medicine, Catholic University of Atma Jaya, Jalan Pluit Raya 2, Jakarta 14440, Indonesia; ^2^Department of Microbiology, Faculty of Medicine, University of Indonesia, Jalan Pegangsaan Timur 16, Jakarta 10320, Indonesia; ^3^Department of Pediatrics, Faculty of Medicine, Neonatal Unit Dr. Cipto Mangunkusumo Hospital, University of Indonesia, Jalan Diponegoro 71, Jakarta 10430, Indonesia

## Abstract

*Acinetobacter baumannii* (*A. baumannii*) is Gram-negative coccobacilli that has emerged as a nosocomial pathogen. Several reports in Indonesia showed the continuous presence of *A. baumannii*. This study aimed to determine the incidence of *A. baumannii* bacteremia in neonates in the Neonatal Unit Dr. Cipto Mangunkusumo Hospital (RSCM), Jakarta, Indonesia, and assess its role in blood stream infection using antibiogram and genotyping by pulsed-field gel electrophoresis (PFGE). Subjects were neonates with clinical sepsis. Blood specimens from the neonates and samples of suspected environment within the Neonatal Unit were cultivated. Antimicrobial resistance profiles were classified for analysis purpose. *A. baumannii* isolates were genotyped by PFGE to determine their similarity. A total of 24 *A. baumannii* were isolated from 80 neonates and the environment during this period of study. Seven isolates from the neonates showed multiple antimicrobial resistance (MDR), and 82% (*n* = 17) of the environment isolates were also MDR. Antibiotype “d” seemed to be predominant (62.5%). PFGE analysis showed a very close genetic relationship between the patients and environment isolates (Dice coefficient 0.8–1.0). We concluded that a mode of transmission of environmental microbes to patients was present in the Neonatal Unit of RSCM and thus needed to be overcome.

## 1. Introduction

*Acinetobacter* spp. are ubiquitous in the environment, that is, soil and water, and occasionally isolated from mucous membrane, secretion, and skin of hospitalized patients, also on surfaces of hospital environment [1]. This aerobic Gram-negative coccobacilli has emerged as important nosocomial pathogen. Clinical sepsis (CSEP) is included in the blood stream infections (BSI) category and restricted only for infant less than 1 year old [2]. However, in protocols of CDC/NHSN 2013, CSEP criteria are not in the list of BSI group but laboratory-confirmed BSI type 1, 2, and 3 [3].

Multidrug resistance of *A. baumannii* has caused morbidity, mortality, and increased patients' length of stay in hospital in many countries [4–6]. Mortality of patient with *Acinetobacter* sp. infection reached 17%–46% [4, 5]. The continuous presence of this environment microorganism from clinical specimens in Jakarta, Indonesia, has been reported [7, 8]. Since *Acinetobacter* sp. is frequently established as part of skin and respiratory flora of hospitalized patients especially with prolonged periods, assessment of *A. baumannii* as etiology of disease or colonization is a particular challenge [9]. Bacterial typing either fenotype or genotype would be very useful in indicating HAI cases, as known widely [10, 11]. A case-control study is also recommended to be used in Hospital/Healthcare-acquired infections investigation [12].

*Acinetobacter* sp. is nonfastidious and easily grows in routine media such as blood agar, MacConkey, and chocolate agar [9, 13]. It looks smooth, opaque, raised, and creamy colony in blood agar and pale or nonlactose fermenter in MacConkey agar [1, 9]. 

This study aimed to determine the incidence of *A. baumannii* bacteremia in neonates in Neonatal Unit, Dr. Cipto Mangunkusumo Hospital (RSCM), Jakarta, Indonesia, and assess its role in blood stream infection using antibiogram and genotyping by pulsed-field gel electrophoresis (PFGE) method.

## 2. Materials and Methods

### 2.1. Subjects and Specimens

This is a cross-sectional study. Subjects of this study were neonates (0–28 days old; birth weight 1,000–2,000 gram) with clinical sepsis, and within 48 hrs or more of being hospitalized, no clear focal infection was detected, using catheter lines, during 9 months period (June 2010–February 2011). Neonates with birth weight 1,000–2,000 g were in focus because of their viability or being supposed to survive with correct treatment and avoid hospital acquired infection. CDC/NHSN surveillance definition of HAI criteria was used [2]. Two blood specimens from two separate venipunctures drawn simultaneously were cultivated in BACTEC PAED bottles; aseptic procedures were strictly applied as prevention of contamination [2, 9].

All microbiology procedures were carried out at Clinical Microbiology Laboratory of Medical Faculty University of Indonesia (CML-FMUI). 

### 2.2. The Environmental Specimens

Samples from the environment were chosen based on observation of suspected hospital staffs, devices, and patients in the same ward. 

### 2.3. Cultivation and Identification

Blood specimens were cultured using BACTEC PAED. Gram stain were carried out on positive bottles and followed by inoculation on to blood agar and MacConkey. Identification was conducted using coagulase, catalase, mannitol, API STREP, and API STAPH for Gram positive bacteria. While for the Gram negative the tests were oxidase, API 20E, and API 20NE [9]. Incubation at 44°C was also performed to distinguish *A. baumannii* from *A. calcoaceticus* [1, 9].

### 2.4. Antimicrobial Susceptibility Testing

Antimicrobial susceptibility tests using disk diffusion method were performed for each isolate. Clinical and Laboratory Standards Institute (CLSI) apply as reference [14]. Antibiotics tested were as follow: ceftazidime, amikacin, trimethoprim-sulfamethoxazole, ampicillin-sulbactam, ofloxacin, levofloxacin, meropenem, imipenem, amoxicillin-clavulanic acid, ceftriaxone, cefotaxime, gentamicin, tobramycin, tetracycline, ciprofloxacin, aztreonam, cefepime, piperacillin-tazobactam, and ticarcillin.

Antibiotype was determined as described by Tega et al., 2007 [11], and marked as a lowercase letter. Differences in one or two antibiotics susceptibility were grouped in the same antibiotypes. 

### 2.5. Storage

All of the isolates were stored in nutrient broth containing glycerol 7.5% (v/v) and kept in −80°C. *A. baumannii* isolates were subcultured on blood agar prior to genotyping.

### 2.6. Genotyping

Plugs containing bacterial pellets were prepared prior to *Apa*I (Vivantis) enzyme digestion. The procedures were conducted as described by Suwanto and Kaplan [15]. Electrophoresis was carried out using CHEF-DR II apparatus (Bio-Rad Laboratories, CA, USA) in TBE buffer 0.5x at 14°C. Initial and final time of electrophoresis was 5.3–34.9 seconds; running time was 19.5 hours. *Rhodobacter sphaeroides* 2.4.1. was used as molecular marker [15]. Analysis was conducted using Unweighted Pair Group Means Method (UPGMA) as described by Seifert et al., 2005, to determine the isolates' pulsotypes [16].

## 3. Results

### 3.1. Subjects and Specimens

Out of 80 neonates included in this study, 28 neonates (35%) were diagnosed with sepsis as confirmed by blood culture results. Among these, 5 neonates had *A. baumannii* bacteremia. Thirty-seven isolates obtained from positive blood cultures were Gram-negative bacteria (see Table 1). A total of 7 *A. baumannii* isolates were found.

### 3.2. Specimens from Environment

A variety of microbes were isolated from the environment. Over 50 suspected surfaces and devices located in the ward were examined. Seventeen isolates of *A. baumannii* were isolated from 17 locations. The following were the origins of those isolates: buttons of ventilator, perineum and gluteus area of different neonates, arm (hand and wrist) area of different neonates, hand of nurse, hand of doctors, plastics covered in porthole of infant incubator, bogota bag on patient who had colostomy, and tap water (see Table 2).

### 3.3. Antimicrobial Susceptibility

**Figure 1:**
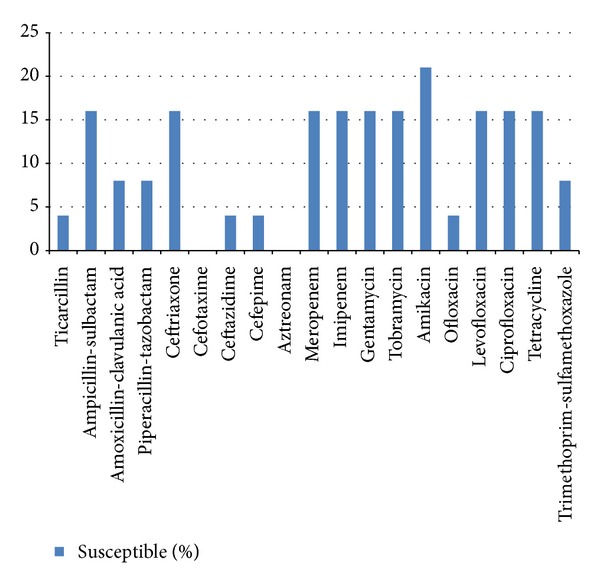
Susceptibility pattern of *A. baumannii* isolates (*n* = 24) to various antibiotics.

A total of 24 *A. baumannii* were tested for their susceptibility to antibiotics. Overall, susceptibility of *A. baumannii* to antibiotics was very low ranging between 0% and 21%. Susceptibility to ceftazidime was 4%, amikacin 21%, trimetoprim-sulfamethoxazole 8%, ampicillin-sulbactam 16%, ofloxacin 4%, levofloxacin 16%, meropenem 16%, imipenem 16%, amoxicillin-clavulanic acid 8%, ceftriaxone 16%, cefotaxime 0%, gentamicin 16%, tobramycin 16%, tetracycline 16%, ciprofloxacin 16%, aztreonam 0%, cefepime 4%, piperacillin-tazobactam 8%, and ticarcillin 4% (Figure 1). All isolates originated from blood showed multiple antimicrobial resistant (MDR), while of the environmental isolates, 82% were MDR. 

Of all *A. baumannii* isolates tested, 9 distinct antibiotypes (“a”–“i”) were determined (Table 2). Predominant antibiotype was “d,” which is composed of 4 isolates that originated from blood and eleven from environment. Thus, 62.5% of the isolates were identical based on their antibiogram profile.

### 3.4. PFGE

**Figure 2:**
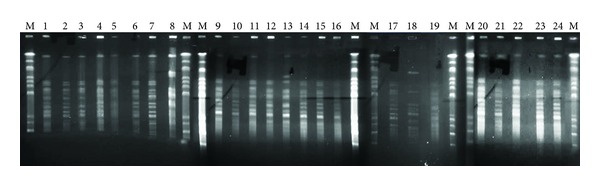
PFGE (Schizotype) profile from *A. baumannii* (*n* = 24) digested by *Apa*I restriction enzyme. Running condition: agarose gel 1.2% in TBE 0.5x, buffer TBE 0.5x, temperature 14°C, ramping pulse 5.3–34.9 second, and run time: 19.5 hours.

**Figure 3:**
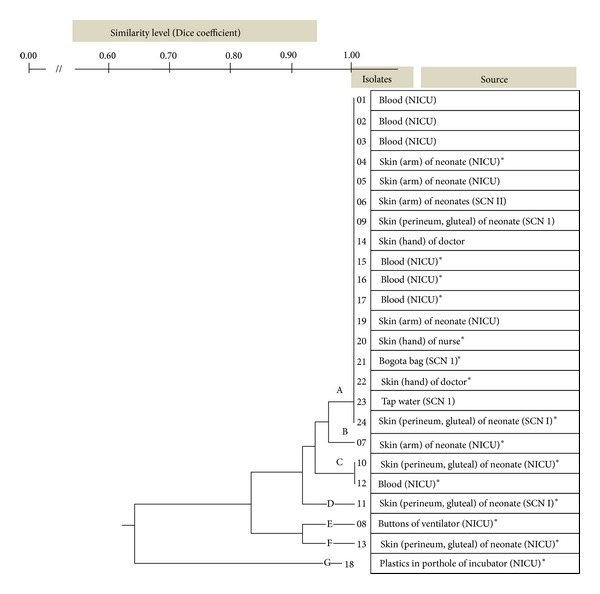
Phenogram of *A.   baumannii* isolates. Seven pulsotypes (“A”–“G”) were identified. Twenty-three isolates (96%) showed a high degree of similarity with Dice coefficient 0.8–1.0, that is, pulsotype “A”–“F”. *Identical antibiotypes.

*Apa*I digested genomic DNA of all 24 *A. baumannii* strains that revealed 11–13 fragments DNA with molecular weight ranges between 63 and 1,100 kb. Estimated genome size varied from 1,663 up to 3,392 kb (Figure 2). The electrophoresis results were transformed into binary data and analyzed by UPGMA software. Seven distinct PFGE types (pulsotype “A”–“G”) were identified (Figure 3). Twenty-three isolates (96%) showed a high degree of similarity with Dice coefficient 0.8–1.0 (pulsotype “A”–“F”); therefore these isolates were very closely related. Of these, 7 isolates were originated from blood and 16 from the environment.

One environmental *A. baumannii* strain, which was isolated from plastics covered in porthole of infant incubator, was not in a close similarity with the other (Dice coefficient 0.667) (Figure 3).

## 4. Discussions

*A. baumannii* is known as environmental microorganism; however it has been found as commensal in human skin, perineum, and digestive system in hospitalized patients. Our findings were in agreement with other reports [15, 17]. Most of *A. baumannii* isolates from the environment in this study derived from skin of the patients themselves indicated that colonization of *A. baumannii* played an important role in the occurrence of hospital infections. The other microbes such as *Staphylococcus aureus, Enterobacter asburiae,* and *Pseudomonas aeruginosa* found as colonization on neonates' skin, plastics covered portholes of incubator, syringe pump, humidifier liquid attached to incubator, and so forth (data not shown), could also give risks of infection when aseptic and antiseptic techniques in invasive procedures were not done properly [13].

Most of *A. baumannii* isolated in the study were multidrug resistant (MDR). According to Abbo et al., 2005, MDR criteria were defined when resistant to all of studied antibiotics, that is, piperacillin-tazobactam, cefepime, ceftazidime, aztreonam, ciprofloxacin, gentamicin, and tobramycin, but could be sensitive to amikacin, ampicillin-sulbactam, imipenem, meropenem, and minocycline [18]. Several reports showed that the usage of broad spectrum antibiotics affected normal flora and induced MDR *A. baumannii* [19, 20]. The same situation occurred in the unit where this study was conducted; broad spectrum antibiotics such as Carbapenem, Piperacillin-Tazobactam, Cefepime, combined with Aminoglycoside, and Amikacin were widely used.

In this study, genetic relationship of *A. baumannii* isolates was assessed using antibiogram and genotyping, while other investigators used only antibiogram for interspecies differentiation, although it was not confirmative [2]. Genotyping by pulsed-field gel electrophoresis has been shown to give better differentiation within species [17, 18]. Our results showed that more than 50% of *A. baumannii* isolates with antibiotype “d” had identical genome profile (pulsotype “A”).

## 5. Conclusions

All* A. baumannii* isolated from blood of neonates with sepsis showed very close genetic relationship to those of environment. We concluded that there was transmission of environmental microbes to patients through contaminated hands of medical staffs and medical equipments. Tracking the agent of nosocomial infection using molecular approach is very fruitful to shed a light on the transmission and resources and therefore would give benefit to infection control.

## Figures and Tables

**Table 1:** Spectrum of microorganisms isolated from blood culture of neonates with sepsis.

Microorganisms	Number of isolates*	Number of neonates
*Acinetobacter baumannii *	7	5
*Pseudomonas aeruginosa *	4	3
*Staphylococcus epidermidis *	3	3
*Enterobacter asburiae *	4	2
*Staphylococcus aureus *	3	2
*Stenotrophomonas maltophilia *	2	2
*Candida tropicalis *	2	1
*Enterobacter cloacae *	2	1
Mould	2	1
*Candida albicans *	1	1
*Candida *sp.	1	1
*Citrobacter freundii *	1	1
*Candida parapsilosis *	1	1
*Enterobacter amnigenus 2 *	1	1
*Enterobacter aerogenes *	1	1
*Klebsiella pneumoniae *	1	1
*Serratia marcescens *	1	1

Total	37	28

*One neonate might experience more than 1 episode of sepsis.

**Table 2:** Antimicrobial Susceptibility patterns of 24 *Acinetobacter baumannii* isolates.

No	Source	AMX	CAZ	CFP	AMK	K	C	TMP/SXT	SAM	OFX	FOS	LVX	MEM	IPM	AMC	CXM	CRO	CTX	CN	TOB	TE	CIP	ATM	FEP	TZP	CSL	TIC	Antibiotype
1	Blood	R	R	R	S	R	R	R	S	R	R	R	R	R	R	R	R	R	R	R	R	R	I	R	R	I	R	a
2	Blood	R	R	R	R	R	R	R	R	I	R	R	S	S	R	R	R	R	R	R	R	R	R	R	R	S	R	b
3	Blood	R	R	R	S	R	R	R	R	R	R	R	R	R	R	R	R	R	R	S	R	R	R	R	R	I	R	c
4	Skin (arm) of neonate	R	R	R	I	R	R	R	R	R	R	I	R	R	R	R	R	R	R	R	R	R	I	R	R	I	R	d
5	Skin (arm) of neonate	S	R	I	S	S	S	R	S	R	S	I	S	S	S	R	I	R	S	S	S	S	I	R	S	I	I	e
6	Skin (arm) of neonate	R	R	R	S	R	R	S	S	I	R	S	S	S	R	R	R	R	R	R	R	R	R	R	I	S	R	f
7	Skin (arm) of neonate	R	R	R	I	R	I	R	R	R	R	I	R	R	R	R	R	R	R	R	R	R	R	R	R	R	R	d
8	Buttons of ventilator	R	R	R	I	R	R	R	I	R	R	I	R	R	R	R	R	R	R	R	R	R	I	R	R	I	R	d
9	Skin (perineum, gluteal) of neonate	R	R	R	R	R	R	R	R	R	R	S	R	R	R	R	R	R	R	R	R	R	R	R	R	I	R	g
10	Skin (perineum, gluteal) of neonate	R	R	R	I	R	R	R	I	R	R	I	R	R	R	R	R	R	R	R	R	R	I	R	R	I	R	d
11	Skin (perineum, gluteal) of neonate	R	R	R	R	R	R	R	I	I	R	I	R	R	R	R	R	R	R	R	R	R	R	R	R	I	R	d
12	Blood	R	R	R	R	R	R	R	R	R	R	I	R	R	R	R	R	R	R	R	R	R	I	R	R	I	R	d
13	Skin (perineum, gluteal) of neonate	R	R	R	I	R	R	R	I	I	R	I	R	R	R	R	R	R	R	R	R	R	R	R	R	I	R	d
14	Skin (hand) of doctor	R	R	R	R	R	R	R	I	R	R	I	R	R	R	R	R	R	R	S	R	R	R	R	R	I	R	h
15	Blood	R	R	R	R	R	R	R	I	R	R	I	R	R	R	R	R	R	R	R	R	R	I	R	R	I	R	d
16	Blood	R	R	R	I	R	R	R	R	I	R	I	R	R	R	R	R	R	R	R	R	R	I	R	R	I	R	d
17	Blood	R	R	R	R	R	R	R	R	R	R	R	R	R	R	R	R	R	R	R	R	R	R	R	R	I	R	d
18	Plastics covered in porthole of infant incubator	R	R	R	R	R	R	R	I	R	R	I	R	R	R	R	R	R	R	R	R	R	R	R	R	I	R	d
19	Skin (arm) of neonate	R	R	R	I	R	R	R	I	I	R	S	R	R	R	R	R	R	R	R	R	R	I	I	R	I	R	g
20	Skin (hand) of nurse	R	R	R	I	R	R	R	I	R	R	I	R	R	R	R	R	R	R	R	R	R	R	R	R	I	R	d
21	Bogota bag	R	R	R	R	R	R	R	R	R	R	R	R	R	R	R	R	R	R	R	R	R	R	R	R	R	R	d
22	Skin (hand) of doctor	R	R	R	I	R	R	R	I	R	R	I	R	R	R	R	R	R	R	R	R	R	R	R	R	I	R	d
23	Tap water	S	S	I	S	S	R	S	S	S	S	S	S	S	S	I	I	I	S	S	S	S	I	S	S	S	S	i
24	Skin (perineum, gluteal) of neonate	R	R	R	R	R	R	R	I	R	R	I	R	R	R	R	R	R	R	R	R	R	R	R	R	I	R	d

CAZ: ceftazidime, CFP: cefoperazone, AMK: amikacin, K: kanamycin, C: chloramphenicol, TMP/SXT: trimethoprim-sulfamethoxazole, OFX: ofloxacin, FOS: fosfomycin, LVX: levofloxacin, MEM: meropenem, IPM: imipenem, AMC: amoxicillin-clavulanic acid, CXM: cefuroxime, CRO: ceftriaxone, CTX: cefotaxime, CN: gentamicin, TOB: tobramycin, TE: tetracycline, CIP: ciprofloxacin, ATM: aztreonam, FEP: cefepime, TZP: piperacillin-tazobactam, CSL: cefoperazone-sulbactam, and TIC: ticarcillin.

S: sensitive, I: intermediate, and R: resistant.

Intermediate result is assumed as resistant result.
